# The Criminal Alliance between the Newspaper and Patent Medicines

**Published:** 1905-11

**Authors:** 


### The Criminal Alliance Between the Newspaper and Patent Medicines.

The current number of *Collier's Weekly* prints an important editorial under the above title. To strengthen its utterances the article is given a wide border made up of half-tone reproduced advertisements clipped from the pages of leading daily papers in several of our most enlightened centers of population. In the same issue of the paper is an editorial which refers to the way in which many of our best cities are systematically infected with typhoid fever—a way which should bring the blush of shame to the face of every city official whose name is on the pay-roll of any of the cities named.

Indeed it is high time that the entire educated American people should rouse from a lethargy, the result of a belief which many have that we are easily the most highly civilized nation on earth. There are many respects in which European countries give us by comparison an appearance which strongly resemble an amount of currency less than a half-dollar, which has long been the proverbial standard of cheapness; and this without making a comparison between the death rate from typhoid fever in the Japanese army and our own mortality during the Spanish-American war.

The patent medicine habit has taken firm hold on the people—they are constant druggers—as Mr. Hapgood suggests, old ladies who would indignantly refuse to partake of beer or any other known alcoholic beverage, habitually sustain their flagging spirits with peruna cocktails or safe-cure toddies. In the creation of this national drug-habituation the newspapers have played a principal part. By direct inference, also, for a large part of the wherewithal to be fed and clothed the newspapers are directly beholden to the proprietors of the death-dealing patent medicine.

Retail druggists are right in denying responsibility for this drug-craving that compels them to sell the stuff—and physicians, who are occasionally blamed, have little or nothing to do with it. The manufacturers and the newspapers are to blame—and it is this class of offenders that laws must reach if the evil is to be mitigated.

Incidentally it might be as well to do two things: To enforce the law regarding the advertising and sale of abortifacients and to encourage in every way those daily papers and other periodicals who refuse to become particeps criminis by carrying such advertising or who join in the holy war against the criminal manufacturer of poison, especially prepared for self-administration.—*Western Medical Review.*

